# Chemoprevention of Urothelial Cell Carcinoma Tumorigenesis by Dietary Flavokawain A in UPII-Mutant Ha-ras Transgenic Mice

**DOI:** 10.3390/pharmaceutics14030496

**Published:** 2022-02-24

**Authors:** Zhongbo Liu, Liankun Song, Jun Xie, Anne R. Simoneau, Edward Uchio, Xiaolin Zi

**Affiliations:** 1 Department of Urology, University of California, Irvine, CA 92868, USA; liuzb727@gmail.com (Z.L.); liankuns@hs.uci.edu (L.S.); xiej@uci.edu (J.X.); asimonea@uci.edu (A.R.S.); euchio@hs.uci.edu (E.U.); 2 Chao Family Comprehensive Cancer Center, University of California, Irvine, CA 92868, USA; 3 Department of Pharmaceutical Sciences, University of California, Irvine, CA 92617, USA

**Keywords:** flavokawain A, urothelial cell carcinoma, UPII-mutant Ha-ras transgenic mice

## Abstract

Non-muscle-invasive bladder cancer (NMIBC) has one of the highest recurrence rates among all solid cancers and the highest lifetime treatment cost per patient. Therefore, the development of chemoprevention strategies for reducing the occurrence and recurrence of NMIBC as well as its burdens on the healthcare system is valuable. Our aim was to determine whether flavokawain A (FKA), a kava chalcone isolated from the kava plant, can target the in vivo activated Ha-ras pathway for prevention and treatment of NMIBC. UPII-mutant Ha-ras transgenic mice that develop papillary urothelial cell carcinoma were fed orally with vehicle control or FKA-formulated food for 6 months starting at 6 weeks of age. Seventy-nine percent (15/19) of male mice fed with 6 g FKA per kilogram (kg) of food survived beyond the 6 months of treatment, while 31.6% (6/19) of control food-fed male mice survived the 6-month treatment period (*p* = 0.02). The mean bladder weights in FKA vs. control food-fed mice were 0.216 ± 0.033 vs. 0.342 ± 0.039 g in male mice (*p* = 0.0413) and 0.043 ± 0.004 vs. 0.073 ± 0.004 g in female mice (*p* < 0.0001); FKA reduced bladder weight by 37% and 41%, respectively. The tumor burdens, determined by the wet bladder weight, in these mice were inversely related to plasma FKA concentrations. In addition to decreased bladder weight, FKA treatment significantly reduced the incidences of hydronephrosis and hematuria. FKA-treated mice exhibited more well-differentiated tumors in the bladder and ureter. Immunohistochemical analysis of FKA-treated tumors compared to those in the control group revealed fewer Ki-67- and survivin-positive cells and an increased number of p27- and TUNEL-positive cells, indicating that FKA inhibits proliferation and induces apoptosis. Overall, the results suggest that FKA can target the in vivo activated Ha-ras pathway for the prevention and treatment of NMIBC.

## 1. Introduction

Bladder cancer is the fourth most common cancer in men and the twelfth most common in women in the United States [1]. The average age at the time of diagnosis is 73 years [2]. About 90% of bladder cancers are urothelial cell carcinomas (previously known as transitional cell carcinomas). Bladder cancer is stratified into non-muscle-invasive bladder cancer (NMIBC) and muscle-invasive bladder cancer (MIBC), which determines treatment options. NMIBC is more common and has one of the highest recurrence rates among all solid cancers. Patients after initial diagnoses of NMIBC often undergo long-term follow-up with repeated, invasive diagnostic and treatment procedures. These procedures not only cause significant pain and compromise quality of life, but also add tremendous financial burdens to patients and their families. NMIBC has the highest lifetime treatment cost per patient, costing over $140,000 per case or ~$4 billion annually in the US alone [3,4]. Therefore, dietary prevention approaches or oral drugs that are effective in preventing and treating the recurrence of NMIBC are an important alternative to improve the quality of patients’ lives and reduce the financial burdens for the management of NMIBC.

Activation of the receptor tyrosine kinase (RTK)-Ras pathway through mutations in the *HRAS* and fibroblast growth factor receptor (FGFR)-3 genes, as well as the overexpression of Ha-ras, FGFRs, HER3, and HER4, occurs in 70–90% of NMIBCs [5,6], and is thought to be a main driver for the tumorigenesis of NMIBC. The urothelium-specific expression of mutant Ha-ras, which is driven by the urothelium-specific uroplakin II (UPKII) promoter, results in the sequential development of simple urothelial hyperplasia, papillary hyperplasia, nodular hyperplasia, and low-grade papillary urothelial cell carcinoma within 6 months of age in our UPII-mutant Ha-ras transgenic mouse model [6]. This model mimics human noninvasive papillary transitional urothelial cell carcinoma (UCC) in both pathology and molecular pathways [5,6], and is therefore appropriate for examining the efficacy of agents for preventing and treating the recurrence and progression of NMIBC.

Flavokawain A (FKA) is a potential chemopreventive agent that we have identified from the kava plant [7]. The root extracts of the kava plant have been consumed as home-made beverages in social activities by Pacific Islanders for centuries [8]. Recently, the products of kava root extracts have gained popularity in clinical and recreational use for their effects on the alleviation of anxiety [9]. We have previously shown that FKA, a predominant chalcone isolated from the kava plant root, is a potent apoptosis inducer against the growth of human urinary bladder cancer cell lines, and preferentially inhibits the growth of p53 mutant cancer cells [10]. In addition, we have reported that dietary administration of FKA-formulated food to UPII-SV40T transgenic mice, a model which produces a functional inactivation of p53/pRb proteins in the mouse bladder, increases the survival of the mice, reduces tumorigenesis, and delays the progression of carcinoma in situ (CIS) to high-grade papillary and muscle-invasive UCC [11]. FKA was also found to be excreted primarily through the urinary tract and concentrated in the urine [11]. Given the well-recognized heterogeneity of human urinary bladder cancer [2,12], here we further examined the chemoprevention potential of FKA against mutant Ha-ras-driven urothelial tumorigenesis. We found that dietary FKA significantly increased the survival of UCC-bearing mice, decreased the tumor burden, and reduced incidences of hydronephrosis and hematuria. Immunohistochemistry (IHC) analysis indicated that dietary FKA reduced cell proliferation and induced apoptosis in tumor tissues through the up-regulation of p27 expression and the down-regulation of survivin.

## 2. Materials and Methods

### 2.1. Breeding and Genotyping

All mice were bred and genotyped as described in our previous publication [6,13]. In brief, heterozygous UPII-mutant Ha-ras+/− females were cross-bred with heterozygous UPII-mutant Ha-ras+/− males to generate offspring. Transgenic pups were confirmed by tail DNA extraction and Southern blotting with a probe located at the 3′ end of the UPII promoter to identify a transgene fragment (1.7 kb) and an endogenous UPII gene fragment (1.4 kb) [6,13]. Homozygous mice with a 1:1 ratio of the transgene to the endogenous UPII gene which had hyperactivation of the Ha-ras oncogene and developed full-blown tumors represented 100% of the mice that were selected for the proposed experiments.

### 2.2. FKA Diet, Experimental Groups, and Animal Care

Six-week-old, homozygous UPII-mutant Ha-ras transgenic mice were fed with vehicle control or with a 0.6% FKA (0.6% FKA (*w*/*w*) in AIN-93M purified) formulated diet for 6 months or until their death. In addition, age-matched nontransgenic mice were administered either the control or the 0.6% FKA diet for the same durations to serve as overall controls. These mice were randomly assigned into different experiment groups with a comparable initial body weight in each group. All diets were prepared commercially (Dyets, Inc., Bethlehem, PA, USA). Mice were permitted free access to food and water. All animals were examined daily for morbidity, mortality, clinical signs of ataxia, and toxicological effects including respiratory depression, neurobehavioral abnormalities, color of skin and eyes (a sign of liver toxicity), and motor activity. Food consumption and animal body weight were recorded bi-weekly. Animal care and treatments were in accordance with Institutional guidelines and the approved protocol by UCI (protocol #:2004-2540).

### 2.3. FKA Measurement

Plasma FKA concentration was measured as described in our previous publication [11]. Briefly, FKA was extracted by acetonitrile and subjected to an Acquity Ultra-Performance Liquid Chromatography (UPLC) system (Waters Corp., Milford, MA, USA) coupled with a Micromass Quattro Micro Liquid chromatography–mass spectrometry (LC/MS/MS) triple quadrupole mass spectrometer (mass range: 2–2000 *m*/*z*) to monitor transition for FKA of *m*/*z* 315 > *m*/*z* 181 and flavokawain B (internal control) of 284 > 181.

### 2.4. Necropsy, Tissue Processing, and Histology Analysis

The mice were sacrificed with 30–70% CO_2_ asphyxiation followed by cervical dislocation. Plasma samples and urine samples were obtained by cardiac puncture and by bladder massage, respectively, at the end of the experiments. Bladder, ureter, and kidney were removed, fixed in formalin, and paraffin-embedded for standard H&E slide preparation and examination. Any evidence of edema, abnormal organ size, or appearance in non-bladder organs was noted. Sections of each urinary bladder, ureter, and kidney tumors were histologically evaluated by a GU pathologist blinded to the experimental groups. Histological lesions were classified into simple hyperplasia (thickened urothelium), papillary hyperplasia (urothelium with multiple undulating folds yet no true papillary fibrovascular cores, more than seven cells in thickness), nodular hyperplasia (neovascularization and rudimentary fibrovascular cores), and low-grade papillary dysplasia (urothelium with increased cell size, nuclear pleomorphism, and hyperchromatism) as described in published papers [6,11,13].

### 2.5. IHC and DeadEnd Colorimetric TUNEL Assay

IHC analysis was performed on paraffin-embedded sections (5 μm thick) using mouse monoclonal anti-Ki-67 antibody (Abcam, 1:800), anti-survivin (Cell Signaling, 1:200), and anti-p27/Kip (BD, 1:100) primary antibodies as described previously. The second antibody staining was performed using biotinylated rabbit anti-mouse IgG (1:200 in 10% normal goat serum) by following R&D systems Cell & Tissue Staining Kit instructions and published papers [11,13]. Negative controls were treated only with PBS under identical conditions. For apoptosis analysis, the DeadEnd Colorimetric TUNEL system (Promega, WI) was used to detect apoptotic cells as described in published papers [11,13]. Proliferating cells and apoptotic cells were quantified by counting the Ki-67-positive cells and the TUNEL-positive cells, respectively, tallying the total number of cells at 12 arbitrarily selected fields at ×200 magnification in a double-blinded manner.

### 2.6. Analysis of Urine

Urine glucose, protein, blood/hemoglobin, specific gravity, pH, leukocytes, nitrite, ketones, urobilinogen, and bilirubin that were measured by Chemstrip 4MD urinalysis test strips (Roche Diagnostics) as described previously [13].

### 2.7. Statistical Analysis

Means, standard deviations, and confidence intervals of all quantitative data were computed using GraphPad Prism statistical software (San Diego, CA, USA). Analysis of variance (ANOVA) or Student’s *t*-test followed by the Bonferroni *t*-test for multiple comparisons was used to compare means of tumor, organ, and body weights between vehicle control and FKA diet groups. The product limit method of Kaplan and Meier and the log-rank test were used to compute and compare survival curves between vehicle control and FKA diet groups. All statistical measures were two-sided, and *p*-values < 0.05 were statistically significant.

## 3. Results

### 3.1. Homozygous UPII-Mutant Ha-ras Mice That Were Fed with a FKA Containing Diet Have a Better Survival than Those Fed with Control Diet

**Figure 1:**
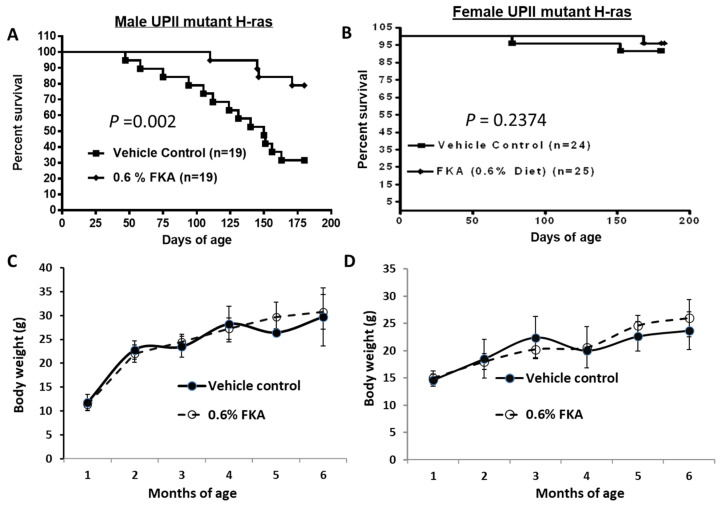
The chemopreventive efficacy of dietary FKA on the survival of UPII-mutant Ha-ras urothelial-tumor-bearing mice. (**A**,**B**) Male and female UPII-mutant Ha-ras mice were fed daily with control diet and diet containing 0.6% FKA starting at 6 weeks of age until their death or up to 6 months of age. (**C**,**D**) The mean body weights were recorded and compared every month between male and female mice fed control diet and 0.6% FKA diet.

To determine the effects of dietary FKA on the full process of mutant Ha-ras-initiated urothelial carcinogenesis, a cohort of the UPII-mutant Ha-ras transgenic mice were genotyped by Southern blotting as described previously and randomized into either the vehicle control diet (AIN-93M) or a modified diet containing 6 g/kg FKA (0.6%) for 6 months [6,11]. Figure 1A shows that about 32% (6/19) and 79% (15/19) of control- and FKA-fed male UPII-mutant Ha-ras mice, respectively, survived for longer than 6 months. The FKA diet increased the survival rate of male UPII-mutant Ha-ras mice by an absolute increase of 47% (log-rank test, *p* = 0.002). The survival rates of female UPII-mutant Ha-ras mice in control and FKA diet groups were about 95% (23/24) and 96% (24/25) at 6 months of age (Figure 1B, *p* = 0.237). Because female mutant Ha-ras mice survived much longer than the males, it was unfeasible to follow up the effect of a FKA diet on the full process of mutant Ha-ras-initiated urothelial carcinogenesis in female mice. In addition, there was no significant difference in body weights between control and FKA-fed mice (Figure 1C,D).

### 3.2. Dietary FKA Inhibits Mutant Ha-Ras Initiated Tumorigenesis in the Bladder of Mice

**Figure 2:**
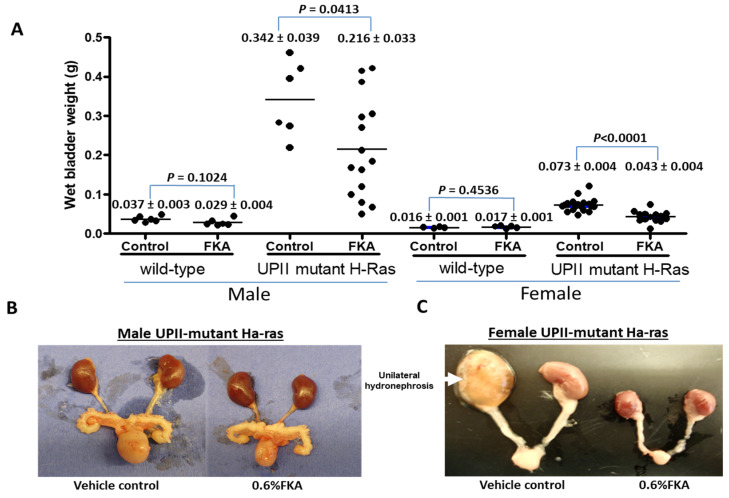
The chemopreventive efficacy of dietary FKA on urothelial tumorigenesis of homozygous UPII-mutant Ha-ras transgenic mice. (**A**) Bladder weights of wild-type and UPII-mutant Ha-ras transgenic mice fed with control diet or diet containing 0.6% FKA for 6 months. Mean bladder weight ± SD. (**B**,**C**) Macroscopic examination revealed enlarged bladders, ureters, and kidneys in control diet groups compared to FKA diet groups in both male and female mice.

We used mouse bladder weight as a surrogate for tumor burden or growth. Compared to mice fed the control diet, the mean bladder weight in male and female UPII-mutant Ha-ras transgenic mice fed the FKA diet was significantly reduced by 36.9% and by 42.2%, respectively (vehicle control vs. FKA diet: male group, 0.3421 ± 0.03927 g (*n* = 6) vs. 0.2157 ± 0.03275 g (*n* = 15), *p* = 0.0413; female group, 0.07331 ± 0.003618 g (*n* = 20) vs. 0.04308 ± 0.003575 g (*n* = 15), *p* < 0.0001 at 6 months of age) (Figure 2A). Male UPII-mutant Ha-ras transgenic mice in control groups developed on average 4.7-fold larger tumors in the bladder than the female transgenic mice. There were no differences in mean bladder weights of wild-type mice without any detectable tumor between mice fed control and FKA diets (*n* = 6 in each group, all *p*-values > 0.05, Figure 2A). We also observed much more enlarged kidneys, ureters, and bladders in UPII-mutant Ha-ras mice fed the control diet compared to those fed the FKA diet (Figure 2B,C).

### 3.3. Dietary FKA Reduces the Incidence of Low-Grade Papillary Carcinomas in Male UPII-Mutant Ha-Ras Transgenic Mice

**Figure 3:**
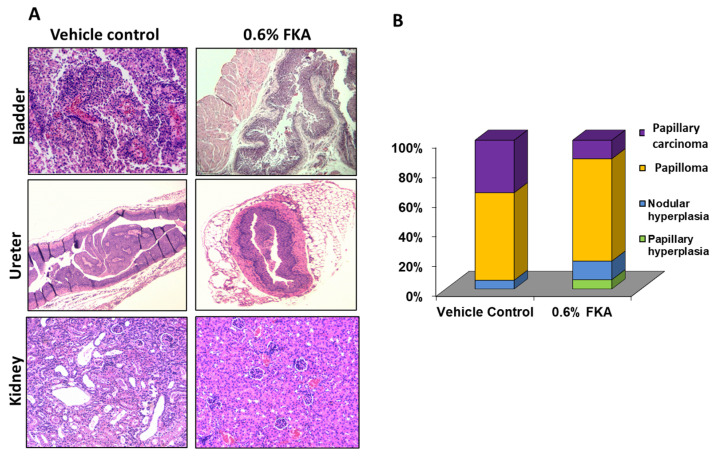
The chemopreventive efficacy of dietary FKA on pathological progression of urothelial carcinogenesis in male UPII-mutant Ha-ras transgenic mice. (**A**) Macroscopic examination of bladders, ureters, and kidneys after male UPII-mutant Ha-ras transgenic mice were fed with control diet or diet containing 0.6% FKA for 6 months. Magnification: 200×. (**B**) Percentages of papillary hyperplasia, nodular hyperplasia, papilloma, and papillary carcinoma after male UPII-mutant Ha-ras transgenic mice were fed with control diet or diet containing 0.6% FKA for 6 months.

Male UPII-mutant Ha-ras transgenic mice fed with FKA-formulated food had fewer incidences of low-grade papillary urothelial carcinomas in the bladder, ureter, and kidney compared to control-diet-fed mice (Figure 3). We assumed the lesion scores of papillary hyperplasia, nodular hyperplasia, papilloma, and papillary carcinoma as 1, 2, 3, and 4 for semi-quantitative scoring, respectively. Then we calculated a composite lesion score of a lesion as a lesion score multiplied by the percentage (%) of the lesion in each bladder. The average sums of the composite lesion scores for vehicle control vs. 0.6% FKA diet were estimated to be 3.21 ± 0.73 (*n* = 15) vs. 1.43 ± 0.32 (*n* = 6) (mean ± SD, *p* < 0.05), respectively. This result indicates that dietary FKA can inhibit the development of early-stage urothelial carcinoma from pre-cancerous lesions.

### 3.4. Dietary FKA Decreases Hematuria and Hydronephrosis

**Figure 4:**
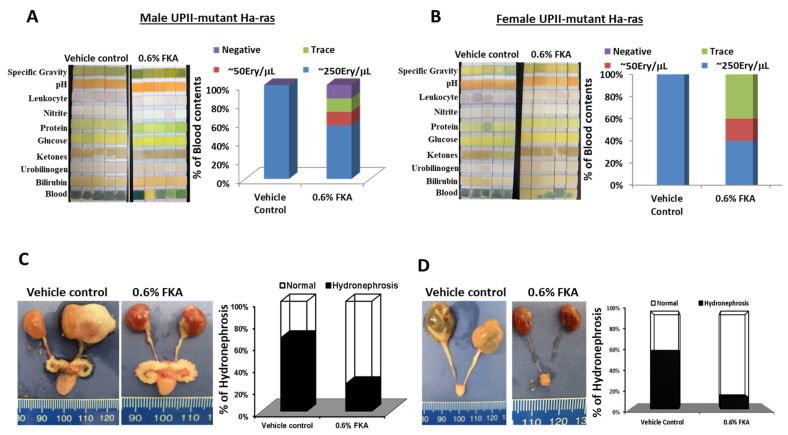
The chemopreventive effect of dietary FKA on hematuria and hydronephrosis. (**A**,**B**) Photographs of dipsticks after loading with urine from male and female homozygous UPII-mutant Ha-ras transgenic mice which were fed with control diet or diet containing 0.6% FKA for 4 months (**left panel**), and percentage of mice with hematuria in control and FKA diet groups (**right panel**). (**C**,**D**) Macroscopic examination showing enlarged kidneys in control-diet-fed mice compared to 0.6% FKA-diet-fed mice (**left panel**), and percentage of mice with hydronephrosis in control and FKA diet groups (**right panel**).

Hematuria is integral in the presentation and subsequent diagnosis of bladder cancer. Approximately 85% of bladder cancer patients seek care due to blood in the urine, and it is the most common symptom of bladder cancer [14]. We have previously shown that the appearance of hematuria in UPII-mutant Ha-ras mice was associated with the initiation of papillary tumor in these mice [13]. Compared to 100% of control-diet-fed mice that demonstrated on urine dip strip about 250 erythrocytes/microliter in urine at 4 months of age, only 20% (2/10) and 0% (0/10) of FKA-diet-fed male and female mice, respectively, reached the level of 250 erythrocytes/microliter by the end of the 4 months (Figure 4A,B). This result suggests that the preventive effect of FKA on bladder cancer development could be monitored by assessing the level of hematuria.

Upper tract urothelial carcinoma (UTUC) is a subset of urothelial cancers that arise in the renal pelvis or the ureter. UTUC can block the ureter or kidney, leading to hydronephrosis and permanent damage of kidney function [15]. The UPII-mutant Ha-ras transgenic model also mimics human UTUC. Figure 4C,D demonstrates that dietary FKA decreased the incidence of hydronephrosis by an absolute decrease of 52% (from 72% (11/15) in the control group to 17% (1/6) in the FKA group) in male UPII-mutant Ha-ras transgenic mice and by 52% (from 60% (14/24) in the control group to 8% (2/25) in the FKA group) in female mice. These results imply that FKA has the utility of preventing the development of UTUC, which is difficult to treat clinically, and oral agents are preferred.

### 3.5. The Bladder Weights (a Surrogate of Tumor Burdens) of FKA-Diet-Fed Mice Are Inversely Related to Plasma FKA Concentrations

**Figure 5:**
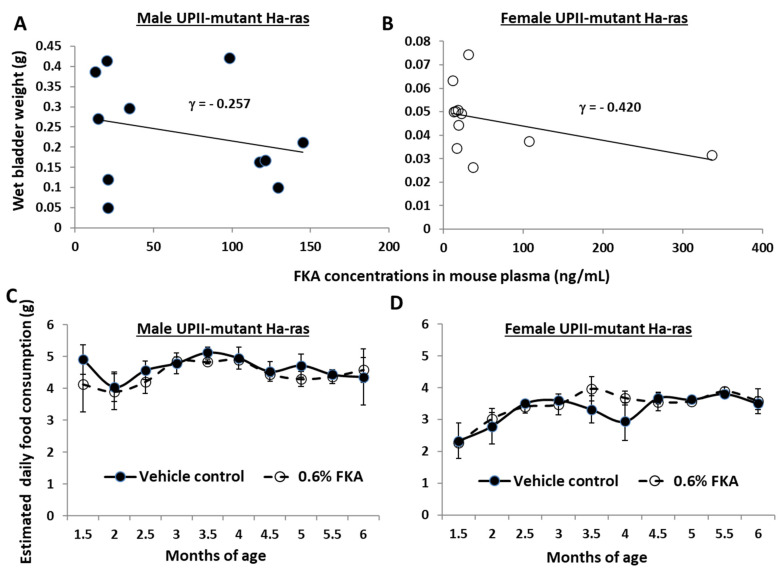
Plasma FKA concentrations are inversely related to tumor burdens. (**A**) Chromatographic separation of FKA was achieved using an ACQUITY UPLC (Waters, UK) with a reversed-phase ACQUITY UPLCTM BEP C18 column (1.7 μm, 2.1 × 50 mm, Waters). FKA was monitored as a precursor ion with an *m*/*z* value of 315 and a fragment ion with a value at 181.14. Correlation analysis between FKA plasma concentrations and bladder weights in male (**A**) and female (**B**) UPII-mutant Ha-ras transgenic mice. The food consumption of male (**C**) and female (**D**) mice was weighted and changed every three days.

Figure 5A,B shows that plasma FKA concentrations were inversely related to the bladder weights of FKA-fed mice, with correlation coefficient values of −0.257 in male mice and −0.420 in female mice (all *p*-values < 0.05). FKA concentrations in the plasma of FKA-fed UPII-mutant Ha-ras transgenic mice ranged from 12.5 to 148.3 ng/mL in males (*n* = 11) and from 15.3 to 346.3 ng/mL in females (*n* = 11), respectively (Figure 5A,B). Female mice appeared to have higher plasma FKA concentrations, which is consistent with a slightly higher efficacy of FKA for reducing tumor burden in the female mice compared to that in the male mice (Figure 2A). There were no differences in food consumption between male and female mice fed FKA diet and control diet (Figure 5C,D).

### 3.6. The In Vivo Anti-Proliferative and Apoptotic Effects of Dietary FKA

**Figure 6:**
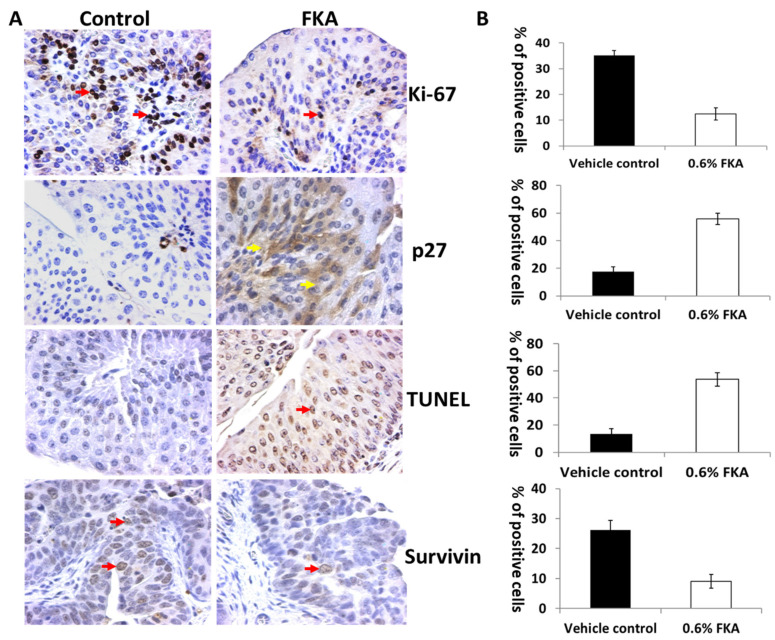
The in vivo mechanisms of dietary FKA in chemoprevention of urothelial carcinogenesis. Bladder tumor sections of 6 randomly selected individual mice from mice fed control or FKA diet for IHC staining of proliferation markers (Ki-67 and p27/Kip1) and apoptotic markers (TUNEL and survivin) expression. (**A**) Representative DAB-stained tissue sections from control- and 0.6% FKA-fed groups showing brown-colored positive cells are depicted at ×200 magnification. (**B**) Positive-staining cells were counted in 12 fields in each group. The percentage of positive cells was calculated and presented as mean ± SD in histography; Student’s *t*-test, all *p*-values < 0.01. Ki-67, TUNEL, and survivin showed nuclear staining (red arrows), whereas p27 staining was in the cytoplasm (yellow arrows).

IHC staining by specific antibodies revealed that there was a decreased level of Ki-67-positive cells and an increased level of p27-positive cells in bladder tumor tissues from FKA-diet-fed mice compared to those from the control diet group (Figure 6A,B: 12.5 ± 2.3% and 35.2 ± 1.8% Ki-67-positive cells for FKA vs. control groups, *p* < 0.01; 55.9 ± 4.1 and 17.0 ± 3.4% p27-positive cells for FKA vs. control groups, *p* < 0.01).

In addition, there were more apoptotic cells present in the bladder tumor tissues of FKA-fed mice (Figure 6A,B: % of TUNEL-positive cells in FKA vs. control groups: 53.7 ± 5.0% and 13.4 ± 4.0%, *p* < 0.01; % of survivin-positive cells in FKA vs. control groups: 9.1 ± 2.3% and 26.2 ± 3.2%, *p* < 0.01). This finding suggests an in vivo anti-proliferative and apoptotic effect of FKA on bladder tumor tissues, thus slowing the progression of bladder cancer.

## 4. Discussion

Bladder cancer evolves through two distinct carcinogenesis pathways: papillary and non-papillary [5,12]. NMIBC accounts for 75–80% of bladder cancers, which progress from hyperplasia to low-grade Ta NMIBC and high-grade Ta/T1 NMIBC through the papillary pathway [5,12]. The papillary pathway is thought to be initiated by RAS mutations, FGFR3 mutations, loss of heterozygosity in chromosome 9, lysine demethylase 6A mutations, or telomerase reverse transcriptase gene-promoter mutations, etc. [5,12]. Frequent recurrence in a short period of time occurs in approximately 50% to 70% of NMIBC cases, which requires long-term surveillance and treatment. The surveillance of NMIBC normally includes cystoscopy every 3–6 months in the first 1–2 years and every year thereafter for 5 years, or lifelong for intermediate- and high-risk patients [16]. This not only imposes large economic burdens on the patients and their families, but also causes many side effects or high morbidity, including pain, risk for infection, and irritation/damage of the urothelium. Therefore, there is a clinical need for the development of low-cost oral drugs for preventing or reducing the recurrence of NMIBC. In this study, we demonstrated that dietary FKA is effective in inhibiting mutant Ha-ras-initiated urothelial tumorigenesis.

Plasma concentrations of FKA were inversely related to tumor burden, suggesting the oral dosage of FKA may play a role in the anti-tumor effect of FKA. However, how FKA is metabolized in vivo and whether FKA metabolites have anti-tumor activity or toxicity remain unclear. In vitro metabolism using human liver microsomes by Zenger et al. [17] revealed that demethylation at C-4 on the B-ring of FKA led to the formation of flavokawain C ( the major phase I metabolite) and monoglucuronides (i.e., FKA-2’-O-glucuronide and (E) -FKA-2′-O-glucuronide), major phase II metabolites. Therefore, further studies are ongoing to confirm or identify the FKA metabolites in vivo and to determine the anti-tumor or toxic effects of these FKA metabolites.

Bladder cancer is a highly heterogeneous and biologically complex disease with distinct clinical and molecular phenotypes and prognosis [2,5,11]. Flat dysplasia and/or CIS are thought to be the precursors of muscle-invasive urothelial carcinoma, and progress through the non-papillary pathway after the gradual accumulation of genetic alterations of TP53 mutations, excision repair 2 mutations, loss of heterozygosity in chromosome 9, and others [2,5,11,18,19]. These genetic alterations sometimes result in the acquisition of invasion potential in high-grade papillary tumors and can be the intersection between the papillary and the non-papillary carcinogenesis pathways [2,5,11,18,19]. We previously reported that FKA preferentially inhibits the growth of high-grade, muscle-invasive bladder cancer cell lines (i.e., T24, UMUC3, HT1376, 5637, and HT1197) harboring TP53 mutations; it was more effective on HT1197 and TCCSUP cell lines harboring TP53 mutations in the tetramerization domain over low-grade and p53 wild-type papillary bladder cancer RT4 cells [10]. The growth-inhibitory mechanisms of FKA are also different between these two types of bladder cancer cells [10]. In low-grade p53 wild-type bladder cancer, FKA increases the expression of cell cycle inhibitors p21 and p27, leading to G1 arrest in cell cycle progression, whereas FKA decreases the expression of Myt1 and Wee1 and increases the expression of cyclin B1, resulting in a G2-M arrest in p53 mutant-type, muscle-invasive, or metastatic bladder cancer cells [10]. Compared to FKA inducing G1 arrest in p53 wild-type MCF-7 cells, the selective cytotoxicity and induction of G2M arrest of FKA to breast cancer cell line MDA-MB231with mutant p53 were also reported by Abu et al. [20]. These results have provided a consistent support for the selectivity of FKA to p53 mutant cancer cells. Therefore, further studies are in progress to investigate the underlying mechanisms or to identify the direct targets of FKA contributing to its selectivity in mutant p53 cells. By comparison (though not presented/reviewed here), the UPII-SV40T transgenic mice mimics the non-papillary pathway with loss of tumor suppressors p53 and pRb functions to develop CIS, high-grade papillary tumors, and high-grade UCC co-existing with muscle-invasive UCC and/or metastasis [11,21]. In a previous paper we reported that 0.6% dietary FKA inhibited tumor growth by weight by 59% in UPII-SV40T male transgenic mice [12]. Here, in a UPII-mutant Ha-ras transgenic mouse model mimicking the low-grade papillary pathway, the mean tumor weight was reduced by 36.9% under the same dose of dietary FKA, but to a lesser extent than was seen in the UPII-SV40T model. In addition, dietary FKA significantly increased the expression of cell cycle inhibitor p27 in tumor tissues. These in vivo findings are consistent with our previously reported in vitro results [10], suggesting that FKA preferably inhibits the growth of p53 defective bladder cancer.

Bladder cancer is associated with multiple abnormalities of genomic and molecular pathways [2,12]. Targeting one genetic or genomic alteration or one pathway by a specific inhibitor may easily result in treatment resistance or escape from preventative effect by bypassing the pathway or the factor. In addition, NMIBC can also progress to MIBC through the non-papillary pathway. Therefore, simultaneous targeting of the papillary pathway (e.g., Ras mutations) and the non-papillary (e.g., p53 mutation) pathways could be a novel treatment or prevention option for patients with NMIBC in the post-operative prevention setting with high risk for disease recurrence and progression to MIBC.

In addition to *RAS* mutations, FGFR-3 mutations and an overexpression of EGFR, HER2, and HER3 to activate the RTK-Ras pathway are also commonly identified in NMIBC [5,6]. We have shown that FKA preferentially inhibited the growth of HER2-overexpressing breast cancer cell lines (i.e., SKBR3 and MCF7/HER2) versus those with less HER2 expression (i.e., MCF7 and MDA-MB-468) through inhibition of Cdc2 and Cdc25C phosphorylation and downregulation of expression of Myt1 and Wee1 [22]. To more efficiently inhibit tumor growth in NMIBC, combination approaches of FKA with RAS-targeting drugs (e.g., sotorasib and salirasib) [23,24], FGFR-3 inhibitor (e.g., erdafitinib) [25], and EGFR inhibitors (e.g., erlotinib and gefitinib) [26,27] should be further explored.

In summary, dietary FKA is effective in inhibiting urothelial tumorigenesis and increasing the survival of tumor-bearing mice in both UPII-mutant Ha-ras and UPII-SV40T transgenic models, which mimic the papillary pathway (e.g., *RAS* mutations) and the non-papillary (e.g., *TP53* mutation) pathways, respectively, leading to recurrence and progression of human urinary bladder cancer. In addition, multiple in vivo chemoprevention studies of dietary FKA have demonstrated its excellent safety in long-term consumption (6 months) without observing any weight loss or organ toxicities [11,28,29]. Therefore, FKA may be worth investigating as an oral agent to prevent or reduce cancer recurrence and progression after transurethral resection of urothelial tumors for a better management of NMIBC.

## Data Availability

Not applicable.
